# Mother–Infant Skin-to-Skin Contact: Short‐ and Long-Term Effects for Mothers and Their Children Born Full-Term

**DOI:** 10.3389/fpsyg.2020.01921

**Published:** 2020-08-28

**Authors:** Ann E. Bigelow, Michelle Power

**Affiliations:** Department of Psychology, St. Francis Xavier University, Antigonish, NS, Canada

**Keywords:** mother–infant skin-to-skin contact, postpartum depressive symptoms, breastfeeding, infants’ self-agency, mother–child emotional communication, mother–child short‐ and long-term relations

## Abstract

This brief report reviews findings from a longitudinal study of skin-to-skin contact (SSC) with mothers and full-term infants and a follow-up study of these dyads when the children were 9 years. Findings infer the positive influence of SSC on mother–child interactions in infancy and into children’s middle childhood. Mothers and infants in SSC and control groups were seen when infants were 1 week, 1 month, 2 months, and 3 months. SSC group mothers reported fewer depressive symptoms in infants’ early weeks and had a greater reduction in salivary cortisol, a physiological stress indicator, in infants’ first month (Bigelow et al., 2012). SSC group mothers who initially chose to breastfeed continued to breastfeed their infants throughout the 3 months, whereas breastfeeding mothers in the control group declined over the visits (Bigelow et al., 2014). When engaged in the Still Face Task with their mothers, SSC group infants showed the still face effect with their affect at 1 month, a month before the control group infants did so (Bigelow and Power, 2012). At 3 months, SSC group infants were social bidding to their mothers during the still face phase. When the children were 9 years, the mother–child dyads engaged in conversations about the children’s remembered emotional events (Bigelow et al., 2018). Mother–child dyads who had been in the SSC group showed more engagement and reciprocity in the conversations than mother–child dyads who had been in the control group. Oxytocin, which is induced by SSC, is hypothesized to be an underlying factor that helped the mother–infant relationship have a positive trajectory with long-term benefits.

## Introduction

This brief report reviews findings from one of the few mother–infant skin-to-skin contact (SSC) studies involving full-term infants that follows mother–infant dyads beyond the immediate post-birth period (Bigelow and Power, 2012; Bigelow et al., 2012, 2014). When the children were 9 years, these dyads participated in the only long-term SSC follow-up study with children born full-term (Bigelow et al., 2018). Findings indicate the facilitating association of SSC to the mother–child relationship in infancy and its sustaining association into children’s middle childhood. Oxytocin, which is released in SSC, is hypothesized to mediate the effects associated with SSC in infancy and beyond.

## Infancy Study

### Introduction

In SSC, the baby, dressed only in a diaper, is placed on the mother’s bare chest so that frontal body contact is skin-to-skin. SSC stimulates oxytocin release in mother and infant (Uvnäs-Moberg et al., 2015). Oxytocin, a hormone and neuropeptide, is associated with calmness, bonding, and stress reduction. The full-body contact and sound of the mother’s heartbeat are thought to simulate sensations that the infant experienced prenatally, thereby further reducing stress (Whitelaw and Sleath, 1985).

SSC has many benefits to infants’ post-birth neuro-physical adjustments. Previous studies show that newborns, whether premature or full-term, who have SSC with their mothers have better and more stable physiological functioning than newborns who do not have SSC. SSC is associated with the regulation of newborns’ temperature, heart rate, respiration, and gastrointestinal adaptation (Charpak et al., 1997; Fohe et al., 2000; Bystrova et al., 2003; Bergman et al., 2004; Ludington-Hoe et al., 2006; Moore et al., 2016). Infants who have SSC sleep better (Messmer et al., 1997; Feldman and Eidelman, 2003), cry less (Christensson et al., 1992; Michelsson et al., 1996), and have less pain reaction to routine hospital procedures (Gray et al., 2000; Cong et al., 2012).

The associations of SSC with newborns’ physiological adjustments are well-documented; however, the influence of SSC on the mother, the infant after the newborn period, and the developing mother–infant relationship is less researched. The infancy study followed mothers and their full-term infants over the infants’ first 3 months, examining the associations of SSC during the infants’ first month with maternal depressive symptoms and physiological stress, mothers’ maintenance of breastfeeding, and infants’ responsiveness to their mothers.

The postpartum period increases women’s depression risk and can elevate physiological stress. Studies show that within 6 weeks after delivery, 20% to 40% of mothers report depressive symptoms (Morris-Rush et al., 2003; McCoy et al., 2006).

Maternal depression affects infants as well as mothers. Depression can cause disturbances in maternal behaviors. Depressed mothers show less sensitivity and responsiveness with their infants. They have reduced engagement and playfulness and more irritability in their maternal interactions (Lovejoy et al., 2000; Field, 2010). In contrast, nondepressed mothers typically are socially responsive to infants. In naturally occurring interactions with young infants, nondepressed mothers tend to imitate infants’ actions, mirroring them in a slightly exaggerated manner (Gergely and Watson, 1996, 1999). Such maternal responsiveness facilitates infants’ developing self-awareness that their behavior has predictable reactions in others (Neisser, 1991). Depressed mothers’ reduced responsive behavior hinders infants’ ability to sense their effect on others, putting them at risk for difficulties in cognitive and social-emotional developments (Bigelow, 1999).

Ample evidence indicates breastfeeding is the optimal source of nourishment for young infants (e.g., World Health Organization, 2004). However, many mothers who begin as breastfeeding mothers do not remain so even through their infants’ early weeks. Most mothers terminating breastfeeding before 6 months do so in the infants’ first month (Millar and Maclean, 2005).

Infants’ responsiveness to others develops through early infancy and reflects their experience with their mothers (Bigelow and Rochat, 2006). The Still Face Task assesses infants’ responsiveness to maternal behavior. In this task, initially developed by Tronick et al. (1978), mothers and infants sit facing each other, and there are three sequential phases: initial interactive phase (mother engages the infant normally); still face phase (mother looks at the infant with a neutral expression and does not talk or touch the infant); and reunion phase (mother engages the infant normally again). If the infant has developed expectations for the mother’s behavior, the still face phase should violate these expectations, and the infant should react differently to the still face phase than to the interactive phases. From about 2 months of age through the first year, infants demonstrate the still face effect: they reduce their attention and/or positive affect in the still face phase compared with the interactive phases (Adamson and Frick, 2003; Mesman et al., 2009).

SSC was hypothesized to be associated with reduced maternal depressive symptoms and physiological stress, mothers’ maintenance of the decision to breastfeed, and acceleration of infants’ responsiveness in the Still Face Task.

### Method

Ninety mother–infant dyads participated in this quasi-experiment. Mothers were recruited before their infants’ birth from perinatal clinics at two hospitals in northeastern Canada, neither of which had SSC as standard care at the time of the study. The clinics distributed a brief written description of the study. Mothers who expressed interest in participating were contacted; the study was explained in more detail, and mothers who were still interested signed consent forms. Hospitals were designated as either the SSC or the control group site. Mid-way through the study, the designations were switched; the SSC site became the control site and vice versa. The switch of recruitment sites did not affect the amount of SSC provided in the SSC or control groups. The demographics of the mothers and infants did not differ between the two sites.

SSC group mothers were requested to provide SSC with their infants for 6 h cumulative throughout the day through the infants’ first week, then 2 h a day until the infants were 1 month. Control group mothers received no request to provide SSC. SSC was described to all mothers; mothers in both groups kept daily records of the amount of SSC they provided.

Home visits occurred when infants were 1 week, 1 month, 2 months, and 3 months. On each visit, (1) mothers completed the Edinburgh Postnatal Depression Scale (Cox et al., 1987; Cox and Holden, 2003); (2) mothers were asked their method of infant feeding: exclusive breastfeeding (breastmilk only), partial breastfeeding (breastmilk + formula/other foods), or not breastfed (formula/other foods only); (3) maternal interactions were assessed during infant feeding, either breastfeeding or bottle-feeding, with the Nursing Child Assessment Feeding Scale (NCAFS; Summer and Spietz, 1994) Caregiver subscale that assesses mothers’ ability to adapt and modify their behavior in response to their infants, which reflects the mother–infant relationship; and (4) a mother–infant Still Face Task was conducted: initial interactive phase (3 min), still face phase (1 min), and reunion phase (2 min); videotapes of the task were coded, by coders blind to the infants’ grouping, for visual attention to the mother and two measures of positive affect: smiles and non-distress vocalizations. In the 1-week and 1-month visits, saliva samples were taken from the mothers and assayed for cortisol, a physiological indicator of stress.

### Results

Table 1 shows the demographics of the mothers and infants in the SSC and control groups and the SSC mothers provided in the infants’ first week and weeks 2 through 4. SSC group mothers provided slightly less SSC than the 6 h requested in the infants’ first week but more than the 2 h requested in weeks 2 through 4, whereas control group mothers provided minimal SSC throughout the infants’ first month.

**Table 1:** Demographics of the mothers and infants in the skin-to-skin contact (SSC) and control groups and SSC hours/day provided in the infants’ first week and weeks 2 through 4.

Mother variables	Skin-to-skin contact group	Control group
Age in years^*^	*M* = 31.7 (*SD* = 5.9)	*M* = 28.3 (*SD* = 4.2)
SES	*M* = 49.9 (*SD* = 14.0)	*M* = 49.7 (*SD* = 11.1)
Education	*M* = 3.4 (*SD* = 0.7)	*M* = 3.1 (*SD* = 0.9)
1 = without high school diploma		
2 = high school diploma only		
3 = some university/post-secondary education		
4 = university degree		
Number of other children	*M* = 1.1 (*SD* = 0.7)	*M* = 1.2 (*SD* = 0.7)
1 = no previous child		
2 = 1 previous child		
3 = 2 or more previous children		
Race/ethnicity	100% non-Hispanic White	98% non-Hispanic White, 2% Asian
Infant variables
Sex	48% male	48% male
Birth weight in grams	*M* = 3,640.9 (*SD* = 439.4)	*M* = 3,608.7 (*SD* = 593.3)
Birth length in centimeters	*M* = 52.6 (*SD* = 2.4)	*M* = 52.5 (*SD* = 2.5)
Apgar at 5 min	*M* = 9.9 (*SD* = 0.3)	*M* = 9.8 (*SD* = 0.6)
SSC provided
Infants’ first week^*^	*M* = 5.03 (*SD* = 1.33)	*M* = 0.46 (*SD* = 0.85)
Infants’ weeks 2 through 4^*^	*M* = 2.66 (*SD* = 1.22)	*M* = 0.15 (*SD* = 0.32)

^*^ SSC and control groups were significantly different (*p* < 0.01).

Socio-economic status (SES) scores of the mother or her partner, whoever had the higher score, on a Canadian index (Blishen et al., 1987). Table adapted from Bigelow et al. (2012, p. 376). Copyright © 2012 with permission from AWHONN, Association of Women’s Health, Obstetric, and Neonatal Nurses.

#### Effects of Skin-to-Skin Contact on Maternal Depressive Symptoms and Physiological Stress (Depression Scores and Salivary Cortisol Levels)

SSC group mothers had lower depression scores (*M* = 4.19, *SD* = 3.32) than control group mothers (*M* = 6.90, *SD* = 4.83) on the 1-week visit, *F*(1, 88) = 7.755, *p* = 0.007, and marginally so, on the 1-month visit, *F*(1, 80) = 3.195, *p* = 0.078 (SSC, *M* = 3.00, *SD* = 3.13; control, *M* = 4.78, *SD* = 4.75; Bigelow et al., 2012).

Mothers’ salivary cortisol in SSC and control groups did not differ at the 1-week or 1-month visits. However, difference scores (salivary cortisol at 1 week minus salivary cortisol at 1 month) showed that SSC group mothers had a greater reduction in salivary cortisol than control group mothers, *F*(1, 75) = 4.172, *p* = 0.045 (Bigelow et al., 2012).

#### Effect of Skin-to-Skin Contact on Infant Feeding and Maternal Interactions (Duration of Breastfeeding and Nursing Child Assessment Feeding Scale Caregiver Subscale Scores)

**Figure 1:**
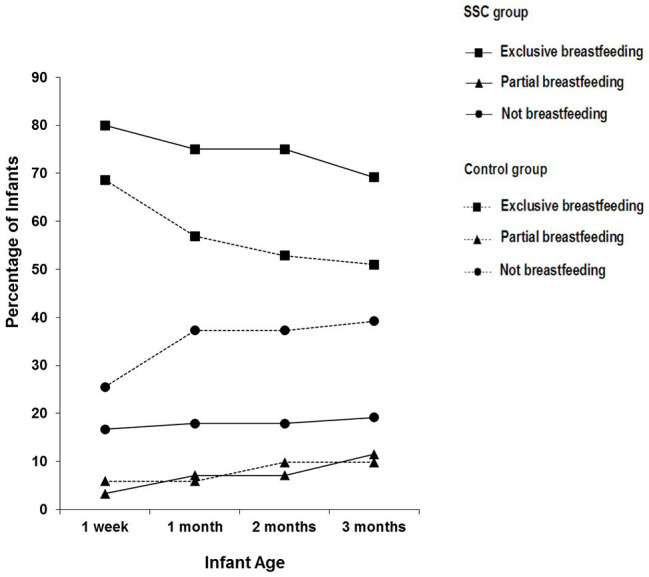
Percentage of infants reported to be exclusively breastfeeding, partially breastfeeding, and not breastfeeding in the SSC and control groups on each of the visits. Reprinted from Bigelow et al. (2014, p. 57). Copyright © 2013 Michigan Association for Infant Mental Health (MAIMH), published by Wiley Periodicals, Inc. Used with permission.

SSC and control group mothers did not differ on their choice to breastfeed, either exclusively or partially, when their infants were 1 week. All the SSC group mothers who began the study as breastfeeding mothers, either exclusively or partially, continued to breastfeed their infants through the 3-month course of the study (Cochran’s *Q*-test, *p* = 1.00), whereas breastfeeding mothers in the control group declined over the visits (Cochran’s *Q*-test, *p* < 0.001; Bigelow et al., 2014; see Figure 1).

Breastfeeding mothers had higher scores on the NCAFS Caregiver subscale, indicating more sensitivity to their infants, when infants were 2 months, *F*(1, 67) = 5.316, *p* = 0.024, and 3 months, *F*(1, 69) = 6.640, *p* = 0.012, than non-breastfeeding mothers (Bigelow et al., 2014). Mothers’ scores did not differ in SSC and control groups.

#### Effect of Skin-to-Skin Contact on Infants’ Responsiveness (Still Face Task)

Mixed analyses of variance, accompanied by trend analyses, were conducted at each infant age for visual attention, smiles, and non-distress vocalizations (Bigelow and Power, 2012).

At 1 week, infants in both groups showed less attention in the still face phase than in the interactive phases, as indicated by the main effect for phase, *F*(2, 150) = 4.764, with no interaction.

**Figure 2:**
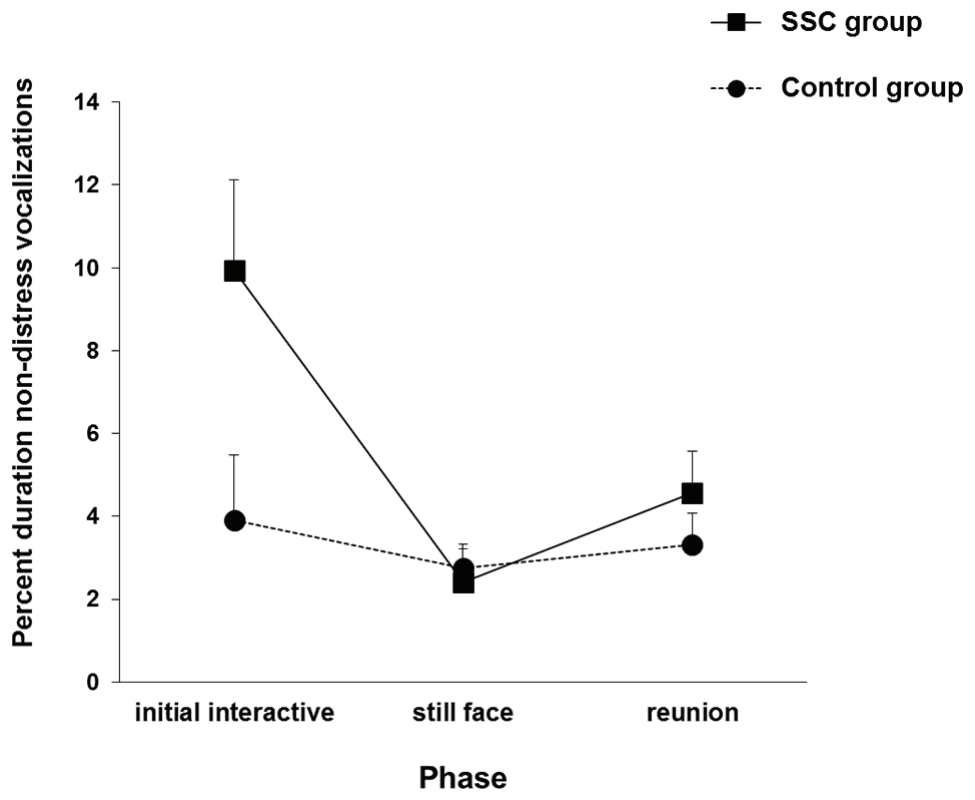
Infants’ non-distress vocalizations in the SSC and control groups during each phase of the Still Face Task at the 1-month visit. Vertical bars represent standard errors. Adapted from Bigelow and Power (2012, p. 245). Copyright © 2012, with permission from Elsevier.

Differences in affect responses between the groups appeared at 1 month. A group × phase interaction for non-distress vocalizations, *F*(2, 152) = 3.854, *p* = 0.023, found that only SSC group infants showed the still face effect (see Figure 2).

By 2 months, infants in both groups showed the still face effect with smiling via a main effect for phase, *F*(2, 146) = 19.983, *p* < 0.001, with no interaction.

**Figure 3:**
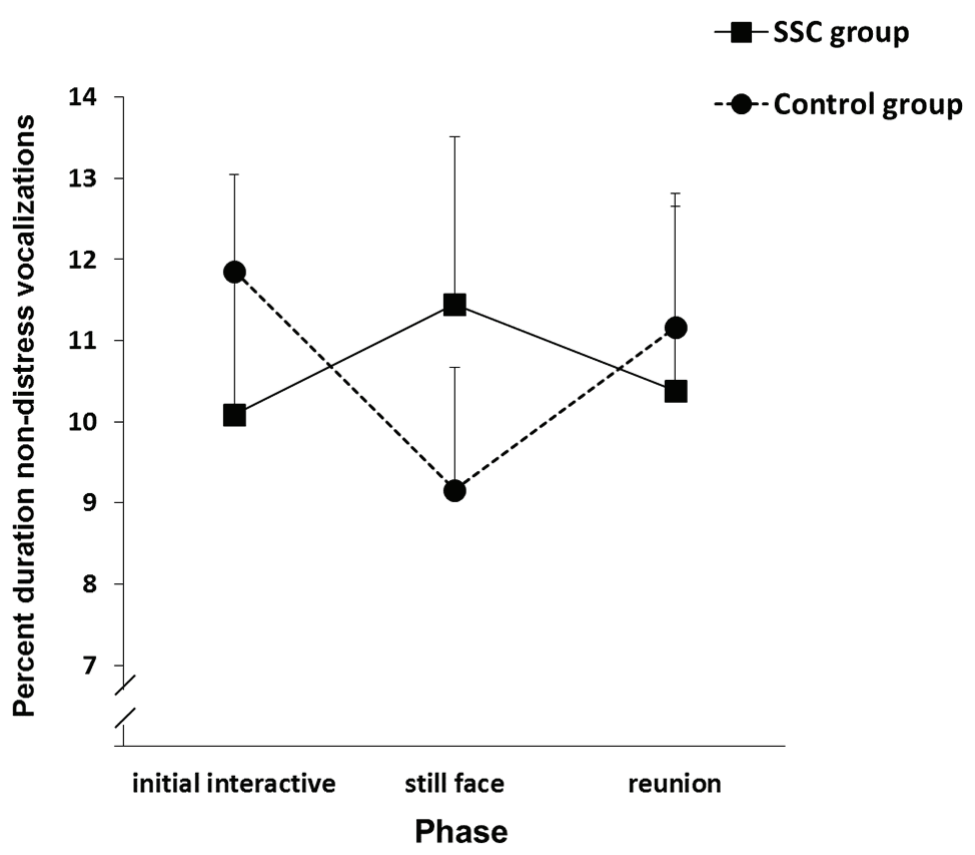
Infants’ non-distress vocalizations in the SSC and control groups during each phase of the Still Face Task at the 3-month visit. Vertical bars represent standard errors. Adapted from Bigelow and Power (2012, p. 247). Copyright © 2012, with permission from Elsevier.

At 3 months, a quadradic trend group × phase interaction in non-distressed vocalizations, *F*(1, 70) = 5.273, *p* = 0.025, revealed that control group infants showed the usual still face effect, but SSC group infants showed the opposite reaction, which is indicative of social bidding to their unresponsive mothers (see Figure 3).

### Discussion

Mothers undergo many transitions in their infants’ first week (Christie et al., 2008). There are physical and hormonal adjustments to being nonpregnant. Mothers who are breastfeeding are also adapting to lactation. Newborn care necessitates many behavioral changes for the mother. First-time mothers are adjusting to motherhood, and the whole family is making accommodations for the newborn. Thus, numerous emotional demands on mothers are concentrated in infants’ early weeks. However, SSC group mothers had fewer depressive symptoms and greater reduction of stress during this time.

Feeding is the context in which the mother–infant relationship is first established. None of the SSC group mothers who began the study as breastfeeding mothers terminated breastfeeding in the course of the study, whereas the percentage of control group breastfeeding mothers declined over the visits. SSC helped mothers maintain their decision to breastfeed, at least through the first 3 months, which is predictive of breastfeeding beyond 3 months.

Mothers’ scores on the NCAFS Caregiver subscale were similar in the SSC and control groups, possibly because the NCAFS is scored on dichotomous variables rather than by frequency or duration of behaviors (Chiu and Anderson, 2009). Nevertheless, SSC was associated with breastfeeding, which in turn was related to enhanced maternal interactions. Breastfeeding, like SSC, releases oxytocin in both mother and infant (Uvnäs-Moberg et al., 2005). Behaviorally, breastfeeding heightens the mutual gaze between mother and infant (Lavelli and Poli, 1998) and enhances maternal touch (Dunn and Richards, 1977). These increased maternal behaviors facilitate infants’ ability to associate their own actions with those of their mothers. This growing self-awareness contributes to infants’ emotional responsiveness to their mothers (Bigelow, 1999).

Infants are sensitive to changes in maternal social behavior even as newborns. Both groups of infants demonstrated this with their attention during the Still Face Task. Few previous studies have conducted the Still Face Task with newborns; however, the studies find that these very young infants show less attention to the still face phase compared with the initial interactive phase (Ellsworth, 1987, cited in Muir and Hains, 1993; Bertin and Striano, 2006; Nagy, 2008).

Young infants’ changes in attention and affect during face-to-face interactions may indicate different understandings (Legerstee and Varghese, 2001; Nadel et al., 2005). Changes in attention during the Still Face Task demonstrate awareness of shifts in the partner’s behavior, whereas changes in affect suggest that infants are reacting to violations of their expectations for emotional sharing.

SSC group infants, but not control group infants, showed affect changes to the Still Face Task at 1 month with non-distress vocalizations. Although smiling is the most commonly used indicator of infants’ positive affect, non-distress vocalizations communicate infants’ interest and readiness to engage and are the main signals that prompt maternal interaction (Papousek, 1989; Hsu and Fogel, 2001; Van Egeren et al., 2001).

By 2 months, both groups of infants began showing the still face effect with their smiling. At this age, infants typically start reacting to the Still Face Task with their affect.

At 3 months, SSC group infants were social bidding to their mothers during the still face phase. Social bids are infrequent before 6 months of age, and if they occur, they are typically short-lived attempts at the start of the still face phase (Tronick et al., 1982; Moore et al., 2001). However, at 3 months, SSC group infants were actively trying to reengage their unresponsive mothers. Such behavior suggests that these infants were aware of themselves as effective instigators of social interaction.

## Role of Oxytocin

Oxytocin, a hormone and neurotransmitter, plays a major role in the developing mother–infant relationship (Uvnäs-Moberg et al., 2005). Oxytocin is produced in the hypothalamus and released into blood circulation and into the brain to induce coordinated responses. Oxytocin reduces stress by decreasing activity in the hypothalamic–pituitary–adrenal axis, thus reducing cortisol levels. Autonomic nervous system activity shifts toward the parasympathetic from the sympathetic part of the system, inducing calmness. When administered externally, oxytocin facilitates positive social interaction and stress reduction (Jonas et al., 2008; Velandia, 2012). Similar reactions are promoted by gentle stimulations of sensory nerves, particularly in the chest area, that activate oxytocin release (Uvnäs-Moberg and Prime, 2013). Oxytocin is increased by touch, gentle pressure, and warmth during SSC, as well as breastfeeding. In SSC, increases in oxytocin in one member of the dyad influences increases in the other, and their responses become synchronized, facilitating social connectedness (Uvnäs-Moberg et al., 2015). The pattern of effects associated with SSC suggests that they are mediated by oxytocin.

For infants, much of the physiological responses to SSC shortly after birth, such as stabilization of temperature, heart rate, respiration, and gastrointestinal functioning (Charpak et al., 1997; Fohe et al., 2000; Bystrova et al., 2003; Bergman et al., 2004; Ludington-Hoe et al., 2006; Moore et al., 2016), are due primarily to oxytocin. The anti-stress effects of oxytocin are also important (Uvnäs-Moberg et al., 2015). Being born is stressful. Although stress during birth is beneficial to the infant, heightened stress post-birth is detrimental. The stress-reducing function of oxytocin when newborns are in SSC calms infants; they cry less and have reduced reaction to pain-inducing procedures (Christensson et al., 1992; Michelsson et al., 1996; Gray et al., 2000; Cong et al., 2012). Oxytocin, induced by SSC, also primes infants for breastfeeding (Uvnäs-Moberg and Prime, 2013).

In mothers, oxytocin bolsters positive mood states and enhances maternal behaviors (Carter, 1998; Uvnäs-Moberg, 2003; Uvnäs-Moberg et al., 2015). Mothers’ oxytocin increases in SSC, which facilitates maternal engagement and reduction of depressive symptoms and stress. Breastfeeding also increases mothers’ oxytocin, inducing milk release from the mammary glands and likely strengthening the effects of SSC (Uvnäs-Moberg and Prime, 2013).

Mothers’ affectionate gaze, touch, and facial and vocal expressions are influenced by underlying physiological processes as well as psychological and social factors that affect infants’ responsiveness to their mothers. The close body contact between mother and infant in SSC promotes mothers’ recognition of, and familiarization with, infants’ emotional cues, and infants’ attentiveness to their mothers’ social overtures. Mothers who are sensitive to infants’ affect cues have more frequent positive interactions with their infants and have infants who are more engaged with their mothers (Legerstee and Varghese, 2001). Maternal sensitive behaviors predict infants’ responsiveness and social bids during the Still Face Task (Tronick et al., 1982; Braungart-Rieker et al., 2001; Mcquaid et al., 2009). The development of the mother–infant relationship is a psycho-biological process in which oxytocin enhances the sensitivity of mother and infant to each other.

## Follow-Up Study 9 Years Later

### Introduction

Short-term SSC follow-up studies show continued positive associations to the mother–infant relationship. SSC provided in the infant’s first hour, an early sensitive period, is associated with increased social interaction and stress reduction in mothers and full-term infants a year later (De Chateau and Wiberg, 1984; Bystrova et al., 2009). Kangaroo Care studies (includes SSC, exclusive breastfeeding, and early hospital discharge) with preterm infants show increases in maternal sensitivity and infant competencies months after SSC ends (Feldman et al., 2002; Ohgi et al., 2002; Bigelow et al., 2010). These findings suggest there are sustaining benefits of SSC beyond early infancy.

However, long-term follow-up studies of SSC are rare. Only three such studies are published (Feldman et al., 2014; Charpak et al., 2017; Bigelow et al., 2018), two of which followed infants born premature. Charpak et al. (2017) followed premature infants with prior Kangaroo Care and matched controls for 20 years. Although the researchers did not specifically assess the mother–child relationship, they speculated that the better social functioning of participants with prior Kangaroo Care was due to their mothers’ increased sensitivity and support. Feldman et al. (2014) followed mothers and their premature infants who had been in SSC and control groups for 10 years. They found that children who had been in the SSC group had better cognitive development, better autonomic nervous system functioning, and mother–child interactions were more reciprocal. Prematurity presents risks to infants’ development. Interventions that stabilize preterm infants in early infancy can have long-term physical and cognitive benefits, which, in themselves, may enhance the mother–child relationship.

A follow-up study of mother–child dyads with children born full-term provides a more conservative test of the long-term association of SSC to the mother–child relationship. When children who had been in the infancy study were 9 years, they and their mothers participated in a study that investigated whether the positive trajectory begun in infancy was evident in the children’s middle childhood (Bigelow et al., 2018). The follow-up study assessed how mother–child conversations about remembered emotional events in the children’s lives assist children to explore and reflect upon their emotional experiences.

Children learn about themselves and others in the context of mother–child interactions. Emotion infuses these interactions from early infancy and throughout childhood (Vygotsky, 1978; Schore, 2001). Mothers are the primary regulators of interactions with their infants by their responses to infants’ nonverbal emotional behaviors (Tronick, 2007). SSC is associated with maternal sensitivity to these behaviors and the promotion of infants’ social engagement with their mothers (Feldman et al., 2002; Bigelow and Power, 2012). As children mature, develop language, and advance in cognitive skills, their nonverbal exchanges with their mothers become conversations in which they play an active role. Nevertheless, mothers tend to guide and structure the conversations (Kuebli et al., 1995). Through the way mothers communicate about emotions, children realize that some emotions are acceptable to explore in detail while others are to be minimized (Oppenheim et al., 2007). Mother–child discussions, especially about children’s experiences, influence how children process their emotional life as well as others’ emotional reactions (Dunn et al., 1991; Laible and Thompson, 1998). By assisting children to form open and regulated dialogs about emotion, mothers enable children to acquire a sense of self as understood and accepted (Bretherton and Munholland, 1999; Thompson, 2000; Walters and Cummings, 2000; Koren-Karie et al., 2008).

### Method

Participants were 45 mother–child dyads who had participated in the SSC infancy study. The attrition rate, due to difficulties locating dyads in the original study, did not differ between the SSC and control groups. Dyads who returned for the follow-up study did not differ from dyads who did not return on children’s sex, racial–ethnic parentage, position in the family, or socio-economic status (SES) at children’s birth. However, returning mothers were older and more educated at the children’s birth than mothers who did not return (Bigelow et al., 2018).

The study utilized the Autobiographical Emotional Events Dialogue (Koren-Karie et al., 2003), in which mother–child dyads discuss remembered events when the child felt happy, sad, scared, and mad. Coders, blind to the dyads’ grouping in infancy, coded the videotaped conversations on seven maternal scales that form a Maternal Sensitive Guidance score capturing the mother’s overall guidance and support of her child in the conversations and seven child scales that form a Child Cooperation and Exploration score assessing the child’s elaborations and willingness to talk about the remembered events.

### Results

Mother–child dyads who had been in the SSC group had higher Maternal Sensitive Guidance scores, *F*(1, 38) = 5.776, *p* = 0.021, and Child Cooperation and Exploration scores, *F*(1, 38) = 5.339, *p* = 0.026, than mother–child dyads who had been in the control group (Bigelow et al., 2018). SSC group dyads had more reciprocal exchanges that were open and accepting of the partner’s perspective. Maternal Sensitive Guidance scores mediated the effect of SSC on Child Cooperation and Exploration scores, indicating the influence of SSC on children’s elaboration and willingness to talk about past emotional events in their lives was primarily through the influence SSC had on mothers’ guidance and support as they engaged their children in the conversations.

### Discussion

Reciprocity in mother–child interactions may be a long-term benefit of SSC applicable to children born premature or full-term, for it is a salient theme found in the 10-year follow-up study of Feldman et al. (2014) with children born premature and in the 9-year follow-up study with children born full-term (Bigelow et al., 2018). Mother–child reciprocity begins in infancy and shows stability within mother–child dyads from childhood to adolescence (Feldman, 2010; Feldman et al., 2013). Reciprocal mother–child dialogs assist children to reflect upon their own understandings as well as on the perspectives of others. Children in middle childhood can be active collaborative partners in conversations concerning emotions they have experienced (Reese et al., 1993). However, the influence of SSC on children’s willingness to engage their mothers in conversations about emotional events in their lives was due to the indirect influence of SSC on their mothers’ supportive guidance through these discussions. Mothers’ openness and acceptance of their children’s emotions provide a safe place for children to reflect upon their feelings, which enables children to better understand their emotional reactions as well as the reactions of others (Fonagy et al., 2002).

SSC in infancy was associated with benefits to the mother–child relationship 9 years later. However, additional variables within children (e.g., temperament), their mothers (e.g., attachment history), and external factors (e.g., parental divorce) can affect the mother–child relationship and should be examined in future SSC follow-up studies. Likewise, future studies would benefit from conducting measurements at intervals to determine whether SSC in infancy shows consistent associations with positive mother–child relations through childhood.

In infancy, oxytocin, stimulated by SSC, may help set the mother–child relationship on a positive trajectory. However, oxytocin may do more than stimulate a nurturing effect at the beginning of the relationship. Repeated exposure to oxytocin through tactile contact and breastfeeding may induce long-term changes in stress reactivity, as has been shown in animal studies (Holst et al., 2002). Early effects of oxytocin may be conditioned to shape sustained benefits at both physiological and behavioral levels (Uvnäs-Moberg et al., 2005; Uvnäs-Moberg and Prime, 2013). Exploration of these possibilities awaits future research.

## Data Availability Statement

The datasets generated for this study are available on request to the corresponding author.

## Ethics Statement

The studies involving human participants were reviewed and approved by St. Francis Xavier University Research Ethics Board. Written informed consent to participate in this study was provided by the participants’ legal guardian/next of kin.

## Author Contributions

AB contributed to the conception and design of the research. AB and MP trained the research assistants. MP organized the database. AB and MP conducted the data analyses. AB and MP contributed to the interpretation of the findings. AB wrote the initial draft of the manuscript. AB and MP contributed to the revision of the initial draft and approved the submission.

### Conflict of Interest

The authors declare that the research was conducted in the absence of any commercial or financial relationships that could be construed as a potential conflict of interest.

## References

[ref1] Adamson L. B. Frick J. E. (2003). The still face: a history of a shared experimental paradigm. Infancy 4, 451–473. 10.1207/S15327078IN0404_01

[ref2] Bergman N. J. Linley L. L. Fawcus S. R. (2004). Randomized controlled trial of skin-to-skin contact from birth versus conventional incubator for physiological stabilization in 1200‐ to 2199-gram newborns. Acta Paediatr. 93, 779–785. 10.1111/j.1651-2227.2004.tb03018.x, PMID: 15244227

[ref3] Bertin E. Striano T. (2006). The still-face responses in newborn, 1.5-, and 3-month-old infants. Infant Behav. Dev. 29, 294–297. 10.1016/j.infbeh.2005.12.003, PMID: 17138285

[ref4] Bigelow A. E. (1999). “Infants’ sensitivity to imperfect contingency in social interaction” in Early social cognition: Understanding others in the first months of life. ed. Rochat P. (Mahwah, NJ: Lawrence Erlbaum), 137–154.

[ref5] Bigelow A. E. Littlejohn M. Bergman N. McDonald C. (2010). The relation between early mother-infant skin-to-skin contact and later maternal sensitivity in South African mothers of low birth weight infants. Infant Ment. Health J. 31, 359–377. 10.1002/imhj.20260, PMID: 28543220

[ref6] Bigelow A. E. Power M. (2012). The effect of mother-infant skin-to-skin contact on infants’ response to the still face task from newborn to three months of age. Infant Behav. Dev. 35, 240–251. 10.1016/j.infbeh.2011.12.008, PMID: 22245110

[ref7] Bigelow A. E. Power M. Gillis D. E. MacLellan-Peters J. Alex M. McDonald C. (2014). Breastfeeding, skin-to-skin contact, and mother-infant interactions over infants’ first three months. Infant Ment. Health J. 35, 51–62. 10.1002/imhj.21424, PMID: 25424406

[ref8] Bigelow A. E. Power M. MacLean K. Gillis D. Ward M. Taylor C. (2018). Mother-infant skin-to-skin contact and mother-child interaction nine years later. Soc. Dev. 27, 937–951. 10.1111/sode.12307

[ref9] Bigelow A. E. Power M. MacLellan-Peters J. Alex M. McDonald C. (2012). Effect of mother-infant skin-to-skin contact on postpartum depression and maternal physiological stress. J. Obstet. Gynecol. Neonatal Nurs. 41, 369–382. 10.1111/j.1552-6909.2012.01350.x, PMID: 22537390

[ref10] Bigelow A. E. Rochat P. (2006). Two-month-old infants’ sensitivity to social contingency in mother-infant and stranger-infant interaction. Infancy 9, 313–325. 10.1207/s15327078in0903_3 33412678

[ref300] Blishen B. R. Carroll W. K. Moore C. (1987). The 1981 socioeconomic index for occupations in Canada. Can. Rev. Sociol. Anthropol. 24, 465–488. 10.1111/j.1755-618X.1987.tb00639.x, PMID: 19046145

[ref11] Braungart-Rieker J. M. Garwood M. M. Powers B. P. Wang X. Y. (2001). Parental sensitivity, infant affect, and affect regulation: predictors of later attachment. Child Dev. 72, 252–270. 10.1111/1467-8624.00277, PMID: 11280483

[ref12] Bretherton I. Munholland K. A. (1999). “Internal working models in attachment relationships” in Handbook of attachment: Theory, research, and clinical application. eds. Cassidy J. Shaver P. R. (New York: Guilford Press), 89–111.

[ref13] Bystrova K. Ivanova I. Edhborg M. Matthiesen A. S. Ransjö-Arvidson A. B. Mukhamedrakhimov R.. (2009). Early contact versus separation: effects of mother-infant interaction one year later. Birth 36, 97–109. 10.1111/j.1523-536X.2009.00307.x, PMID: 19489802

[ref14] Bystrova K. Widström A. M. Matthiesen A. S. Ransjö-Arvidson A. B. Welles-Nyström B. Wassberg C.. (2003). Skin-to-skin contact may reduce negative consequences of “the stress of being born”: a study on temperature in newborn infants, subjected to different ward routines in St. Petersburg. Acta Paediatr. 92, 320–326. 10.1080/08035250310009248, PMID: 12725547

[ref15] Carter S. C. (1998). Neuroendocrine perspectives on social attachment and love. Psychoneuroendocrinology 23, 779–818. 10.1016/S0306-4530(98)00055-9, PMID: 9924738

[ref16] Charpak N. Ruiz-Pelaez J. G. Figueroa de Calume Z. Charpak Y. (1997). Kangaroo mother versus traditional care for newborn infants <2000 grams: a randomized, controlled trial. Pediatrics 100, 682–688. 10.1542/peds.100.4.682, PMID: 9310525

[ref17] Charpak N. Tessier R. Ruiz J. G. Hernandez J. T. Uriza F. Villegas J.. (2017). Twenty-year follow-up of Kangaroo mother care versus traditional care. Pediatrics 139:e20162063. 10.1542/peds.2016-2063, PMID: 27965377

[ref18] Chiu S. H. Anderson G. C. (2009). Effect of early skin-to-skin contact on mother-preterm infant interaction through 18 months: randomized controlled trial. Int. J. Nurs. Stud. 46, 1168–1180. 10.1016/j.ijnurstu.2009.03.005, PMID: 19361802 PMC2818078

[ref19] Christensson K. Siles C. Moreno L. Belaustequi A. De La Fuente P. Lagercrantz H.. (1992). Temperature, metabolic adaptation and crying in healthy full-term newborns cared for skin-to-skin or in a cot. Acta Paediatr. 81, 488–493. 10.1111/j.1651-2227.1992.tb12280.x, PMID: 1392359

[ref20] Christie J. Poulton B. C. Bunting B. P. (2008). An integrated mid-range theory of postpartum family development: a guide for research and practice. J. Adv. Nurs. 61, 38–50. 10.1111/j.1365-2648.2007.04464.x, PMID: 18034818

[ref21] Cong X. Cusson R. M. Walsh S. Hussain N. Ludington-Hoe S. M. Zhang D. (2012). Effects of skin-to-skin contact on autonomic pain response in preterm infants. J. Pain 13, 636–645. 10.1016/j.jpain.2012.02.008, PMID: 22595172

[ref22] Cox J. Holden J. (2003). Perinatal mental health: A guide to the Edinburgh Postnatal Depression Scale. London: Gaskell.

[ref23] Cox J. L. Holden J. M. Sagovsky R. (1987). Detection of postnatal depression: development of the 10-item Edinburgh Postnatal Depression Scale. Br. J. Psychiatry 150, 782–786. 10.1192/bjp.150.6.782, PMID: 3651732

[ref24] De Chateau P. Wiberg B. (1984). Long-term effect on mother-infant behavior of extra contact during the first hour postpartum: follow-up at one year. Scand. J. Soc. Med. 12, 91–103. 10.1177/140349488401200205, PMID: 6463623

[ref25] Dunn J. Brown J. Beardsall L. (1991). Family talk about feeling states and children’s later understanding of others’ emotions. Dev. Psychol. 27, 448–455. 10.1037/0012-1649.27.3.448

[ref26] Dunn J. B. Richards M. P. M. (1977). “Observations on the developing relationship between mother and baby in the neonatal period” in Studies in mother-infant interaction. ed. Schaffer H. R. (New York: Academic Press), 427–455.

[ref27] Feldman R. (2010). The relational basis of adolescent adjustment: trajectories of mother-child interactive behaviors from infancy to adolescence shape adolescents’ adaptation. Attach. Hum. Dev. 12, 173–192. 10.1080/14616730903282472, PMID: 20390528

[ref28] Feldman R. Bamberger E. Kanet-Maymon Y. (2013). Parent-specific reciprocity from infancy to adolescence shapes children’s social competence and dialogical skills. Attach. Hum. Dev. 15, 407–423. 10.1080/14616734.2013.782650, PMID: 23544455

[ref29] Feldman R. Eidelman A. (2003). Mother-infant skin-to-skin contact (Kangaroo Care) accelerates autonomic and neurobehavioral maturation in preterm infants. Dev. Med. Child Neurol. 45, 274–281. 10.1017/s0012162203000525, PMID: 12647930

[ref30] Feldman R. Eidelman A. I. Sirota L. Weller A. (2002). Comparison of skin-to-skin (kangaroo) and traditional care: parenting outcomes and preterm infant development. Pediatrics 110, 16–26. 10.1542/peds.110.1.16, PMID: 12093942

[ref31] Feldman R. Rosenthal Z. Eidelman A. I. (2014). Maternal-preterm skin-to-skin contact enhances child physiological organization and cognitive control across the first 10 years of life. Biol. Psychiatry 75, 56–64. 10.1016/j.biopsych.2013.08.012, PMID: 24094511

[ref32] Field T. (2010). Postpartum depression effects on early interactions, parenting, and safety practices: a review. Infant Behav. Dev. 33, 1–6. 10.1016/j.infbeh.2009.10.005, PMID: 19962196 PMC2819576

[ref33] Fohe K. Kropf S. Avenarius S. (2000). Skin-to-skin contact improves gas exchange in preterm infants. J. Perinatol. 20, 311–315. 10.1038/sj.jp.7200378, PMID: 10920790

[ref34] Fonagy P. Gergely G. Jurist E. L. Target M. (2002). Affect regulation, mentalization, and the development of the self. New York: Other Press.

[ref35] Gergely G. Watson J. (1996). The social biofeedback theory of parental affect-mirroring: the development of emotional self-awareness and self-control in infancy. Int. J. Psychoanal. 77, 1181–1212. 10.4324/9780429471643-7, PMID: 9119582

[ref36] Gergely G. Watson J. (1999). “Early socio-emotional development: contingency perception and the social-biofeedback model” in Early social cognition: Understanding others in the first months of life. ed. Rochat P. (Mahwah, NJ: Lawrence Erlbaum), 101–136.

[ref37] Gray L. Watt L. Blass E. M. (2000). Skin-to-skin contact is analgesic in healthy newborns. Pediatrics 105:e14. 10.1542/peds.105.1.e14, PMID: 10617751

[ref38] Holst S. Uvnäs-Moberg K. Petersson M. (2002). Postnatal oxytocin treatment and postnatal stroking of rats reduce blood pressure in adulthood. Auton. Neurosci. 99, 85–90. 10.1016/S1566-0702(02)00134-0, PMID: 12241092

[ref39] Hsu H. Fogel A. (2001). Infant vocal development in a changing mother-infant communication system. Infancy 2, 87–109. 10.1207/S15327078IN0201_6 33451227

[ref40] Jonas W. Wiklund I. Nissen E. Ransjő-Arvidson A. B. Uvnäs-Moberg K. (2008). Influence of oxytocin or epidural analgesia on personality profile in breastfeeding women: a comparative study. Arch. Womens. Ment. Health 11, 335–345. 10.1007/s00737-008-0027-4, PMID: 18726143

[ref400] Koren-Karie N. Oppenheim D. Carasso A. E. Haimovich Z. (2003). Autobiographical emotional events dialogues: Coding manual. University of Haifa, Israel. PMID:

[ref41] Koren-Karie N. Oppenheim D. Getzler-Yosef R. (2008). Shaping children’s internal working models through mother-child dialogues: the importance of resolving past maternal trauma. Attach. Hum. Dev. 10, 465–483. 10.1080/14616730802461482, PMID: 19016053

[ref42] Kuebli J. Butler S. Fivush R. (1995). Mother-child talk about past emotions: relations to maternal language and child gender over time. Cognit. Emot. 9, 265–283. 10.1080/02699939508409011

[ref43] Laible D. J. Thompson R. A. (1998). Attachment and emotional understanding in preschool children. Dev. Psychol. 34, 1038–1045. 10.1037/0012-1649.34.5.1038, PMID: 9779749

[ref44] Lavelli M. Poli M. (1998). Early mother-infant interaction during breast‐ and bottle-feeding. Infant Behav. Dev. 21, 667–684. 10.1016/S0163-6383(98)90037-6

[ref45] Legerstee M. Varghese J. (2001). The role of maternal mirroring on social experiences in three-month-old infants. Child Dev. 72, 1301–1313. 10.1111/1467-8624.00349, PMID: 11699672

[ref46] Lovejoy M. C. Graczyk J. M. O’Hare E. Neuman G. (2000). Maternal depression and parenting behavior: a meta-analytic review. Clin. Psychol. Rev. 20, 561–592. 10.1016/S0272-7358(98)00100-7, PMID: 10860167

[ref47] Ludington-Hoe S. M. Lewis T. Morgan K. Cong X. Anderson L. Reese S. (2006). Breast and infant temperature with twins during shared Kangaroo Care. J. Obstet. Gynecol. Neonatal Nurs. 35, 223–231. 10.1111/j.1552-6909.2006.00024.x, PMID: 16620248 PMC1890034

[ref48] McCoy S. J. Beal J. M. Shipman S. B. Payton M. E. Watson G. (2006). Risk factors for postpartum depression: a retrospective investigation at 4-weeks postnatal and a review of the literature. J. Am. Osteopath. Assoc. 106, 193–198. 10.7556/jaoa.2006.106.4.193, PMID: 16627773

[ref49] Mcquaid N. E. Bibok M. B. Carpendale J. I. M. (2009). Relation between maternal contingent responsiveness and infant social expectations. Infancy 14, 390–401. 10.1080/15250000902839955, PMID: 32693537

[ref50] Mesman J. van IJzendoorn M. H. Bakermans-Kranenburg M. J. (2009). The many faces of the still-face paradigm: a review and meta-analysis. Dev. Rev. 29, 120–162. 10.1016/j.dr.2009.02.001

[ref51] Messmer P. R. Rodriguez S. Adams J. Wells-Gentry J. Washburn K. Zabaleta I.. (1997). Effect of kangaroo care on sleep time for neonates. Pediatr. Nurs. 23, 408–414. 10.18535/jmscr/v5i4.181, PMID: 9282055

[ref52] Michelsson K. Christensson K. Rothganger H. Winberg J. (1996). Crying in separated and non-separated newborns: sound spectrographic analyses. Acta Paediatr. 85, 471–475. 10.1111/j.1651-2227.1996.tb14064.x, PMID: 8740308

[ref53] Millar W. J. Maclean H. (2005). Breastfeeding practices. Health Rep. 16, 23–31.16190322

[ref54] Moore E. R. Bergman N. Anderson G. C. Medley N. (2016). Early skin-to-skin contact for mothers and their healthy newborn infants. Cochrane Database Syst. Rev. 11:CD003519. 10.1002/14651858.CD003519.pub4, PMID: 27885658 PMC6464366

[ref55] Moore G. A. Cohn J. F. Campbell S. B. (2001). Infant affective responses to mother’s still face at 6 months differentially predict externalizing and internalizing behaviors at 18 months. Dev. Psychol. 37, 706–714. 10.1037/0012-1649.37.5.706, PMID: 11552765

[ref56] Morris-Rush J. K. Freda M. C. Bernstein P. S. (2003). Screening for postpartum depression in an inner-city population. Am. J. Obstet. Gynecol. 188, 1217–1219. 10.1067/mob.2003.279, PMID: 12748483

[ref57] Muir D. W. Hains S. M. J. (1993). “Infant sensitivity to perturbations in adult facial, vocal, tactile, and contingent stimulation during face-to-face interactions” in Developmental neurocognition: Speech and face processing in the first year of life. eds. Boysson-Bardies B. de Schonen S. de Jusczyk P. McNeilage P. Morton J. (Dordrecht, NL: Kluwer Academic).

[ref58] Nadel J. Soussignan R. Canet P. Libert G. Gerardin P. (2005). Two-month-old infants of depressed mothers show mild, delayed and persistent change in emotional state after non-contingent interaction. Infant Behav. Dev. 28, 418–425. 10.1016/j.infbeh.2005.03.005

[ref59] Nagy E. (2008). Innate intersubjectivity: newborns’ sensitivity to communication disturbance. Dev. Psychol. 44, 1779–1784. 10.1037/a0012665, PMID: 18999338

[ref60] Neisser U. (1991). Two perceptually given aspects of the self and their development. Dev. Rev. 11, 197–209. 10.1016/0273-2297(91)90009-D

[ref61] Ohgi S. Fukuda M. Moriuchi H. Kusumoto T. Akiyama T. Nugent J. K.. (2002). Comparison of kangaroo care and standard care: behavioral organization, development and temperament in healthy low-birth-weight infants through one year. J. Perinatol. 22, 374–379. 10.1038/sj.jp.7210749, PMID: 12082472

[ref62] Oppenheim D. Koren-Karie N. Sagi-Schwartz A. (2007). Emotion dialogues between mothers and children at 4.5 and 7.5 years: relations with children’s attachment at 1 year. Child Dev. 78, 38–52. 10.1111/j.1467-8624.2007.00984.x, PMID: 17328692

[ref63] Papousek M. (1989). Determinants of responsiveness to infant vocal expression of emotional state. Infant Behav. Dev. 12, 507–524. 10.1016/0163-6383(89)90030-1

[ref64] Reese E. Haden C. A. Fivush R. (1993). Mother-child conversations about the past: relationships of style and memory over time. Cogn. Dev. 8, 403–430. 10.1016/S0885-2014(05)80002-4

[ref65] Schore A. N. (2001). Effects of a secure attachment relationship on right brain development, affect regulation, and infant mental health. Infant Ment. Health J. 22, 7–66. 10.1002/1097-0355(200101/04)22:1<7::AID-IMHJ2>3.0.CO;2-N

[ref66] Summer G. Spietz A. (1994). NCAST caregiver/parent interaction feeding manual. NCAST Publications, Seattle: University of Washington, School of Nursing.

[ref67] Thompson R. A. (2000). The legacy of early attachments. Child Dev. 71, 145–152. 10.1111/1467-8624.00128, PMID: 10836568

[ref68] Tronick E. (2007). The neurobehavioral and social-emotional development of infants and children. New York, NY: W.W. Norton.

[ref69] Tronick E. Als H. Adamson L. Wise S. Brazelton T. B. (1978). The infant’s response to entrapment between contradictory messages in face-to-face interaction. J. Am. Acad. Child Psychiatry 17, 1–13. 10.1016/S0002-7138(09)62273-1, PMID: 632477

[ref70] Tronick E. Z. Ricks M. Cohn J. F. (1982). “Maternal and infant affective exchange: patterns of adaptation” in Emotion and interaction: Normal and high-risk infants. eds. Field T. Fogel A. (Hillsdale, NJ: Erlbaum), 83–100.

[ref71] Uvnäs-Moberg K. (2003). The oxytocin factor: Tapping the hormone of calm, love, and healing. Cambridge, MA: Da Capo Press.

[ref72] Uvnäs-Moberg K. Arn I. Magnusson D. (2005). The psychobiology of emotion: the role of the oxytocinergic system. Int. J. Behav. Med. 12, 59–65. 10.1207/s15327558ijbm1202_3, PMID: 15901214

[ref73] Uvnäs-Moberg K. Handlin L. Petersson M. (2015). Self-soothing behaviors with particular reference to oxytocin release induced by non-noxious sensory stimulation. Front. Psychol. 5:1529. 10.3389/fpsyg.2014.01529, PMID: 25628581 PMC4290532

[ref74] Uvnäs-Moberg K. Prime D. K. (2013). Oxytocin effects in mothers and infants during breastfeeding. Infant 9, 201–206.

[ref75] Van Egeren L. A. Barratt M. S. Roach M. A. (2001). Mother-infant responsiveness: timing, mutual regulation, and interactional context. Dev. Psychol. 37, 684–697. 10.1037/0012-1649.37.5.684, PMID: 11552763

[ref76] Velandia M. (2012). Parent-infant skin-to-skin contact studies: parent-infant interaction and oxytocin levels during skin-to-skin contact after cesarean section and mother-infant skin-to-skin contact as treatment for breastfeeding problems. (PhD dissertation). Solna, Sweden: Karolinska Institutet.

[ref77] Vygotsky L. (1978). Mind in society: The development of higher psychological processes. Cambridge, MA: Harvard University Press.

[ref78] Walters E. Cummings E. M. (2000). A secure base from which to explore close relationships. Child Dev. 71, 164–172. 10.1111/1467-8624.00130, PMID: 10836570

[ref79] Whitelaw A. Sleath K. (1985). Myth of the marsupial mother: home care for very low birth weight infants in Bogota, Colombia. Lancet 325, 1206–1208. 10.1016/S0140-6736(85)92877-6, PMID: 2860400

[ref80] World Health Organization (2004). Promoting proper feeding for infants and young children. Geneva: World Health Organization.

